# Hsa_circ_0001806 promotes glycolysis and cell progression in hepatocellular carcinoma through miR‐125b/HK2

**DOI:** 10.1002/jcla.23991

**Published:** 2021-10-19

**Authors:** Xueyi Chen, Pengyun She, Caihua Wang, Lina Shi, Tieying Zhang, Yanfei Wang, Haixia Li, Lu Qian, Man Li

**Affiliations:** ^1^ College of Life Sciences Northwest University Xi’an China; ^2^ The First Affliliated Hospital of Xi’an Jiao Tong University Xi’an China; ^3^ Department of Endocrinology The Affiliated Hospital of Northwest University Xi’an NO.3 Hospital Xi’an China; ^4^ Department of Neurology The Affiliated Hospital of Northwest University Xi’an NO.3 Hospital Xi’an China; ^5^ Department of Geriatrics Xianyang first people’s Hospital Xianyang China; ^6^ Xi'an Key Laboratory of Cardiovascular and Cerebrovascular Diseases The Affiliated Hospital of Northwest University Xi’an NO.3 Hospital Xi’an China; ^7^ Department of Internal Medicine The Affiliated Hospital of Northwest University Xi’an NO.3 Hospital Xi’an China

**Keywords:** glycolysis, hepatocellular carcinoma, HK2, hsa_circ_0001806, miR‐125b

## Abstract

**Objective:**

Hepatocellular carcinoma (HCC) is one of the most common malignant tumours and a leading cause of cancer death. Circular RNA (circRNA) has been demonstrated to play an important role in regulating tumour development. The current study aims to explore the specific role of hsa_circ_0001806 during HCC progression.

**Methods:**

The expression of hsa_circ_0001806 in HCC tissues and cells was measured through qRT‐PCR. Cell proliferation, apoptosis and migration were measured using CCK‐8 and Annexin V/PI staining kits, and Transwell assay. Bioinformatics prediction and dual‐luciferase reporter assay were adopted to explore the mechanism underlying the cell function of hsa_circ_0001806 in HCC cells. In addition, glycolysis was assessed by measuring the glucose uptake, lactate production and ATP level using a glucose assay kit, fluorometric lactate assay kit and ATP detection assay kit.

**Results:**

Hsa_circ_0001806 was up‐regulated in HCC tissues and cells and positively associated with the advanced TNM stage, metastasis and poor overall survival. The overexpression of hsa_circ_0001806 promoted HCC cell proliferation, migration and glycolysis and inhibited cell apoptosis, while the silence of hsa_circ_0001806 showed an opposite effect. Furthermore, hsa_circ_0001806 acted as a sponge of miR‐125b to up‐regulate hexokinase II (HK2) expression. In addition, the inhibition of miR‐125b and HK2 overexpression partly reversed the inhibitory effect of hsa_circ_0001806 silencing on HCC cell proliferation, migration and glycolysis.

**Conclusion:**

The inhibition of hsa_circ_0001806 suppressed HCC cell proliferation, migration and glycolysis through mediating miR‐125b/HK2 axis.

## Graphical abstract

Circ_0001806 is up‐regulated in HCC tissues and cells and was positively associated with the advanced TNM stage, metastasis and worse overall survival. Overexpression of circ_0001806 promoted HCC cell proliferation, migration and glycolysis through acting as a sponge of miR‐125b, up‐regulating HK2 expression. Inhibition of miR‐125b or overexpression of HK2 partly reversed the inhibitory effect of circ_0001806 silencing on HCC cell proliferation, migration and glycolysis.

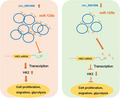

## 1 INTRODUCTION

Hepatocellular carcinoma (HCC) is one of the most common malignant primary tumours occurred in liver and the third leading cause of cancer death. There were about 782,000 cases diagnosed and 746,000 deaths in 2012.^1^ Most patients were diagnosed as HCC at an advanced stage with no obvious symptoms found in the early stages.^2^ According to statistics, the 5 years survival rates of patients with early stage of HCC reached 40% ~70% following radical surgery, while the survival time for patients with advanced liver cancer was only 6–18 months.^3^ In addition, a high recurrence rate was also presented in HCC patients.^4^ Chronic hepatitis B and C, cirrhosis, non‐alcoholic fatty liver disease, excessive alcohol intake and diabetes were reported to be crucial factors leading to HCC.^5^, ^6^, ^7^ Besides, gene alteration was also involved in the tumorigenesis of HCC.^8^ Hence, it is necessary to determine the underlying mechanism by which HCC tumorigenesis is regulated and identify new biomarkers and targets for HCC diagnosis and therapy.

First, circular RNAs (circRNAs) are discovered in 1976 and found to be more stable than long non‐coding RNA.^9^ A covalently closed continuous loop structure without 5′‐caps or 3′‐tails is formed within circRNAs by back‐splicing at exon or intron regions.^10^ Increasing evidence indicated that circRNAs played an important role in tumour evolution and progression.^11^, ^12^ The biological regulatory mechanisms of circRNAs include working as sponges of miRNAs, interacting with RNA‐binding proteins (RBPs), serving as transcriptional and translational regulators, affecting the splicing of pre‐mRNAs and being translated into peptides.^12^, ^13^, ^14^ Based on that, large amount of circRNAs had been regarded as essential potential biomarkers of various tumour diseases.^15^ In addition, growing evidence indicated that numerous circRNAs were dysregulated in HCC tissues and associated with HCC progression^13^, ^16^, ^17^ and could act as competing endogenous RNAs (ceRNAs) to participate tumour progression.^17^ For example, circRNA‐104718 functioned as a ceRNA of miR‐218 to promote HCC progression by regulating thioredoxin domain‐containing protein 5.^12^ Hsa_circ_0001806 derives from centrosome and spindle pole associated protein 1 (CSPP1), located on chromosome 8 (68018139 ~ 68028357). It was also found that hsa_circ_0001806 was dramatically up‐regulated in colorectal cancer tissues and positively associated with tumour node metastasis (TNM) stage, invasion and lymphatic metastasis.^18^ Meanwhile, hsa_circ_0001806 enhanced the stemness of colorectal cancer cells by activating the miR‐193a‐5p/COL1A1 axis.^18^ Recently, evidence discovered an increased expression of hsa_circ_0001806 in HCC tissues and cells.^16^ However, the specific function and mechanism of hsa_circ_0001806 in HCC is still unclear.

In this study, we verified the alteration of hsa_circ_0001806 in HCC tissues and cells, and its relevance with the survival outcomes of HCC patients. Additionally, the role of hsa_circ_0001806 in HCC cell viability, apoptosis, migration and glycolysis was explored. A potential mechanism in which hsa_circ_0001806 exerted on HCC cells was investigated. Overall, our study indicated that hsa_circ_0001806 may act as a potential target for HCC prognosis and therapy.

## 2 MATERIALS AND METHODS

### 2.1 Collection of human HCC tissues

Sixty‐three pairs of HCC tumour tissues and corresponding adjacent normal tissues were collected from HCC patients in Affiliated Hospital of Northwest University Xi'an NO.3 Hospital, from 2012 to 2015. All experiments involving human participants were approved by the Ethics Committee of the Affiliated Hospital of Northwest University Xi'an NO.3 Hospital. All patients provided the written informed consents before tissue collection. The overall survival curve was drawn using the Kaplan‐Meier method and analysed by the log‐rank test according to hsa_circ_0001806 expression (values were expressed as median).

### 2.2 Cell culture

Human normal liver cells, LO2 and HCC cells lines (HepG2, Hep3B and Huh7) were acquired from the Institute of Biochemistry and Cell Biology at the Chinese Academy of Sciences. Cells were cultured in DMEM (Hyclone, Salt Lake City, UT) supplemented with 10% foetal bovine serum (Invitrogen) and 1% penicillin/streptomycin (Gibco). Cells were incubated in a cell culture incubator at 37°C in 5% CO_2_ atmosphere.

### 2.3 Plasmid construction and stable transfection

Overexpression vector (ov) of hsa_circ_0001806 and HK2, small interfering RNA (siRNA) of hsa_circ_0001806, miR‐125b mimic, miR‐125b inhibitor and negative controls (NC) were synthesized and constructed by GenePharma. Cell transfection was performed by using Lipofectamine 3000 (Invitrogen) according to the manufacturer's protocol. Transfection efficiency was confirmed by qRT‐PCR and Western blot at 48 h after transfection.

### 2.4 RNA preparation and qRT‐PCR

Total RNA was isolated using TRIzol reagent (Invitrogen). The obtained RNA samples were quantified on a Nanodrop platform (Thermo Scientific Fisher) and purified using miRNEasy spin columns (QIAGEN). To detect hsa_circ_0001806 expression, the RNA extract was incubated with RNase R (Epicenter, Madison) before RNA purification. Then, RNA was reverse‐transcribed using HiScript II Q RT SuperMix for qPCR (Vazyme). qRT‐PCR was performed using the AceQ SYBR Green PCR Master Mix (Vazyme, Nanjing, China) on the ABI 7900 HT sequence detection system (Applied Biosystems). The circRNA and mRNA levels were normalized to U6 and GAPDH levels. Triplicates of each experiment were performed. The primer sequences used in this study are provided: hsa_circ_0001806, forward: 5'‐CCATCCCATCAGTTCATCCT‐3' and reverse: 5'‐TTCACCTCCAAAGAGCATCC‐3'; Hexokinase 2 (HK2), forward: 5'‐GAGCCACCACTCACCCTACT‐3' and reverse, 5'‐CCAGGCATTCGGCAATGTG‐3'; GAPDH, forward: 5'‐TGTGGGCATCAATGGATTTGG‐3' and reverse: 5'‐ACACCATGTATTCCGGGTCAAT‐3'; miR‐125b, forward: 5′‐TCCCTGAGACCCTAACTTGTGA‐3′ and reverse: 5′‐AGTCTCAGGGTCCGAGGTATTC‐3′; U6, forward: 5′‐TGCGGGTGCTCGCTTCGGCAGC‐3′ and reverse: 5′‐CCAGTGCAGGGTCCGAGGT‐3′. The final quantization of target genes was performed using 2^−ΔΔCt^ method.

### 2.5 Dual‐luciferase reporter assay

The wild type (WT) and mutant type (MUT) of hsa_circ_0001806 luciferase reporter plasmids were constructed and co‐transfected with miRNA mimics into Hep3B cells using Lipofectamine 3000 (Thermo Fisher Scientific). The NC mimic was adopted as a negative control. After being cultured for 48 h, a Dual‐Luciferase Reporter System was used to measure the Firefly and Renilla luciferase activities following the manufacturer's instructions (Promega).

### 2.6 Cell proliferation assay

Hep3B cells (3000 cells/well) were planted into 96‐microwell plates and cultured overnight. After being transfected with overexpression vectors or siRNAs for 48 h, 10 μL of CCK‐8 assay reagent (Dojindo Corp) was added and incubated for another 2 h. The absorbance value was measured at 450 nm in a microplate reader (Thermo Fisher Scientific, Inc.). This experiment was repeated in triplicate.

### 2.7 Cell apoptosis assay

Transfected Hep3B cells (1 × 10^5^ cells/ml) were planted into six‐well plates. After being cultured for 48 h, cells were harvested using a cell scraper and collected into an EP tube. Following washed by PBS twice, cells were double‐stained with Annexin V and propidium iodide (PI) (BD Biosciences Pharmingen) and analysed on an EPICS XL‐MCL flow cytometer (Beckman Coulter, Brea). Each experiment was independently repeated three times.

### 2.8 Cell migration test

Cell migration was evaluated through the Transwell assay. The cultured cells (5 × 10^4^ cells) in 300 ml serum free medium were planted onto the upper chamber (8 μm pore size, Corning). The lower chamber was supplemented with 600 ml medium and 10% FBS. After being cultured for 48 h, cells migrated to the lower chamber were fixed with 4% paraformaldehyde for 15 min and stained with 0.1% crystal violet. The representative images were obtained under an upright microscope, and the migrated cells were counted in 5 randomly selected fields per chamber. This experiment was repeated in triplicate.

### 2.9 Western blot analysis

Cells were harvested and lysed by the treatment with RAPI lysis buffer at 4°C for 30 min. After being centrifuged at 4°C with a speed of 25,764 *g* for 10 min, the supernatant was obtained and the protein concentration was analysed using a BCA Protein Assay Kit (Beyotime). Then, the equal amount of protein samples was separated by 10% sodium dodecyl sulphate‐polyacrylamide gel electrophoresis (SDS‐PAGE) and transferred onto polyvinylidene fluoride (PVDF) membranes (Millipore). After blocking with 5% non‐fat milk, the membranes were incubated with primary rabbit anti‐HK2 antibody (ab209847, 1:1000, Abcam, Inc. Cambridge) at 4°C overnight. Then, membranes were probed with goat anti‐rabbit secondary antibody (ab7090, 1:2000, Abcam). Tubulin (AB6160, 1:2000, Abcam) was used as normalization control. The protein signals were detected under a chemiluminescence system and analysed by FluorChem FC3 Software (ProteinSimple) for quantification.

### 2.10 Glucose uptake, lactate production, and ATP levels

The glucose uptake, lactate production and ATP levels were quantified by using the glucose assay kit (Sigma‐Aldrich), the fluorometric lactate assay kit (Abcam) and Luminescent ATP Detection Assay (Abcam) following the manufacturers' instructions.

### 2.11 Statistical analysis

Statistical analyses were carried out using GraphPad Prism (GraphPad). Student's *t* test was used to compare the statistical significance of differences between two groups. Survival curves for HCC patients were analysed using the Kaplan‐Meier method and log‐rank test. Data were presented as mean ± standard deviation (SD). *P* < 0.05 indicated that the difference was statistically significant.

## 3 RESULTS

### 3.1 Hsa_circ_0001806 was up‐regulated in HCC tissues and cells and correlated with the poor prognosis of patients

**FIGURE 1:**
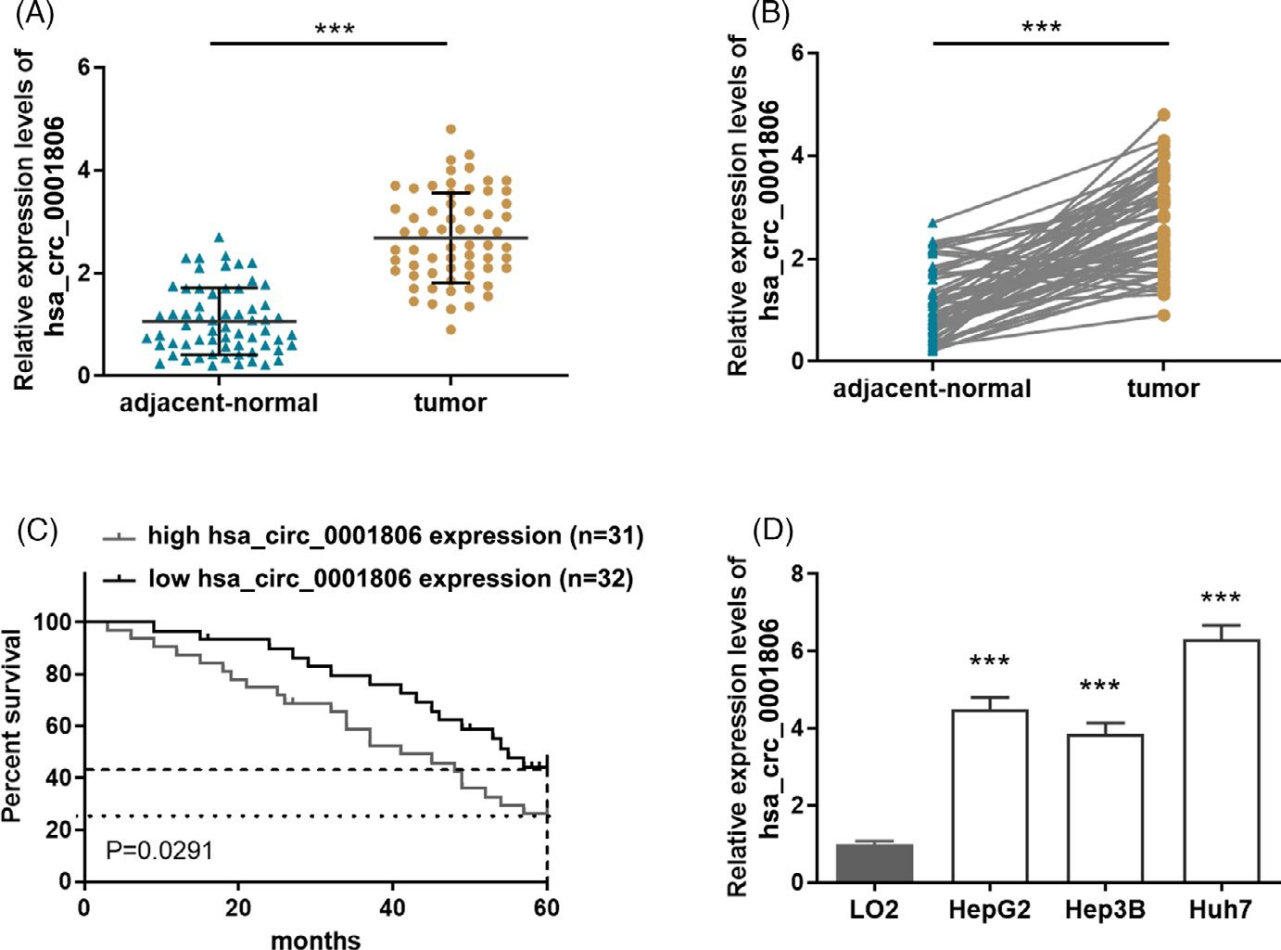
Hsa_circ_0001806 was up‐regulated in HCC tissues and correlated with the poor prognosis. (A and B) The expression of hsa_circ_0001806 in HCC tissues and adjacent normal tissues (*n* = 63). (C) Kaplan‐Meier analysis of a correlation between hsa_circ_0001806 expression and the five‐year survival of HCC patients (Values are expressed as median ± D). *** *p* < 0.001. (D) The expression levels of hsa_circ_0001806 in normal LO2 cells and HCC cells (HepG2, Hep3B and Huh7) were measured through RT‐PCR. ***p < 0.001

As shown in Figure 1A,B, hsa_circ_0001806 was up‐regulated in HCC tissues compared with paired adjacent normal tissues. The clinicopathological characteristics of HCC patients suggested that the high expression of hsa_circ_0001806 was associated with advanced TNM stage (*p* = 0.0273), lymph gland metastasis (*p* = 0.0109) and organ metastasis (*p* = 0.0142) in HCC patients (Table 1). Meanwhile, prognosis analysis calculation suggested that the high level of hsa_circ_0001806 expression was positively associated with lower survival rate (*p* = 0.0291) (Figure 1C). In addition, we found that hsa_circ_0001806 was notably up‐regulated in HCC cells (HepG2, Hep3B and Huh7), compared with LO2 cells (Figure 1D).

**TABLE 1:** Correlation between hsa_circ_0001806 levels in HCC patients and their clinicopathologic characteristics

Clinicopathologic parameters	Low hsa_circ_0001806 (*n* = 32)	High hsa_circ_0001806 (*n* = 31)	*p*‐value
Age (year)			0.5126
≤40	15	12	
>40	17	19	
Gender			
Female	11	7	0.3002
Male	21	24	
Tumour size			0.111
≤5	19	11	
>5	13	20	
TNM stage			0.0273*
Stage I + II	25	16	
Stage III + IV	7	15	
Lymph gland metastasis			0.0109*
No	28	19	
Yes	4	13	
Organ metastasis			0.0142*
No	25	15	
Yes	7	16	

The significance level (p) was determined by chi‐square, *p < 0.05.

### 3.2 Effect of hsa_circ_0001806 on cell viability, apoptosis, migration, and glycolysis in Hep3B cells

**FIGURE 2:**
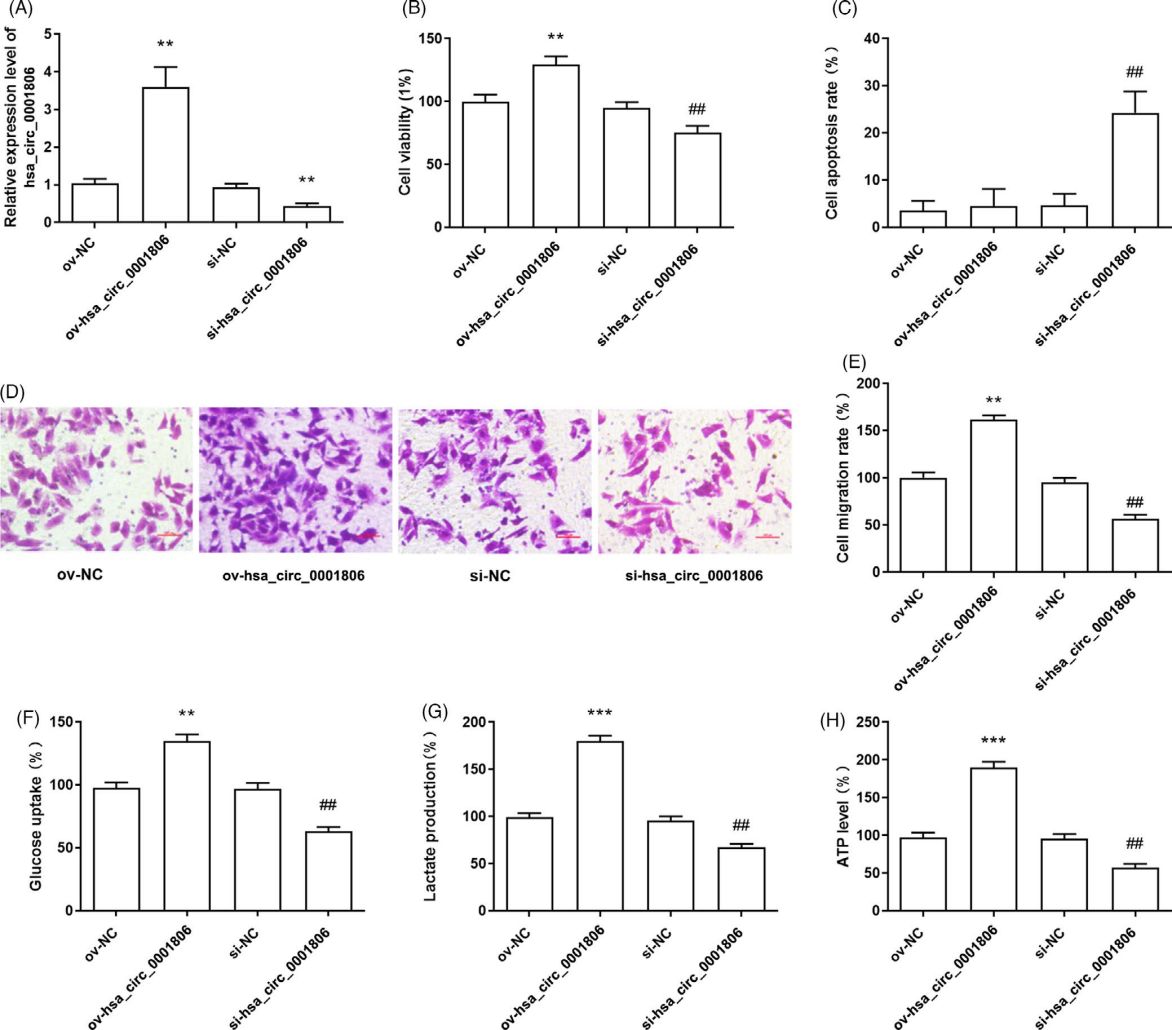
The regulation of hsa_circ_0001806 on cell viability, apoptosis, migration and glycolysis in Hep3B cells. (A) The transfection efficiency of overexpression vector and siRNA of hsa_circ_0001806 in Hep3B cells were determined through RT‐PCR. (B‐E) Cell proliferation, apoptosis and migration were measured using CCK‐8 assay, Annexin V‐FITC Apoptosis Detection kit and Transwell assay. (F‐H) Glucose uptake, lactate and ATP production were detected. **p < 0.01, ***p < 0.001, compared with ov‐NC transfected group; ^##^ *p* < 0.01, compared with si‐NC transfected group

To detect the functional effects of hsa_circ_0001806 on the cell processes of HCC, hsa_circ_0001806 expression was significantly depressed by siRNA (si‐hsa_circ_0001806) and up‐regulated by hsa_circ_0001806 overexpression vector (ov‐hsa_circ_0001806) in Hep3B cells (Figure 2A). The results of CCK‐8 assay indicated that hsa_circ_0001806 overexpression promoted cell viability of Hep3B cells, while the knockdown of hsa_circ_0001806 suppressed the cell viability of Hep3B cells (Figure 2B). Subsequently, we found that hsa_circ_0001806 inhibition significantly induced cell apoptosis in Hep3B cells (Figure 2C). In addition, the result of the Transwell assay showed that the overexpression of hsa_circ_0001806 facilitated Hep3B cell migration, while the silencing of hsa_circ_0001806 suppressed cell migration (Figure 2D,E). What is more, glucose uptake, lactate production and ATP level were significantly elevated by hsa_circ_0001806 overexpression and inhibited by hsa_circ_0001806 inhibition in Hep3B cells (Figure 2F–H).

### 3.3 Hsa_circ_0001806 served as a sponge of miR‐125b

**FIGURE 3:**
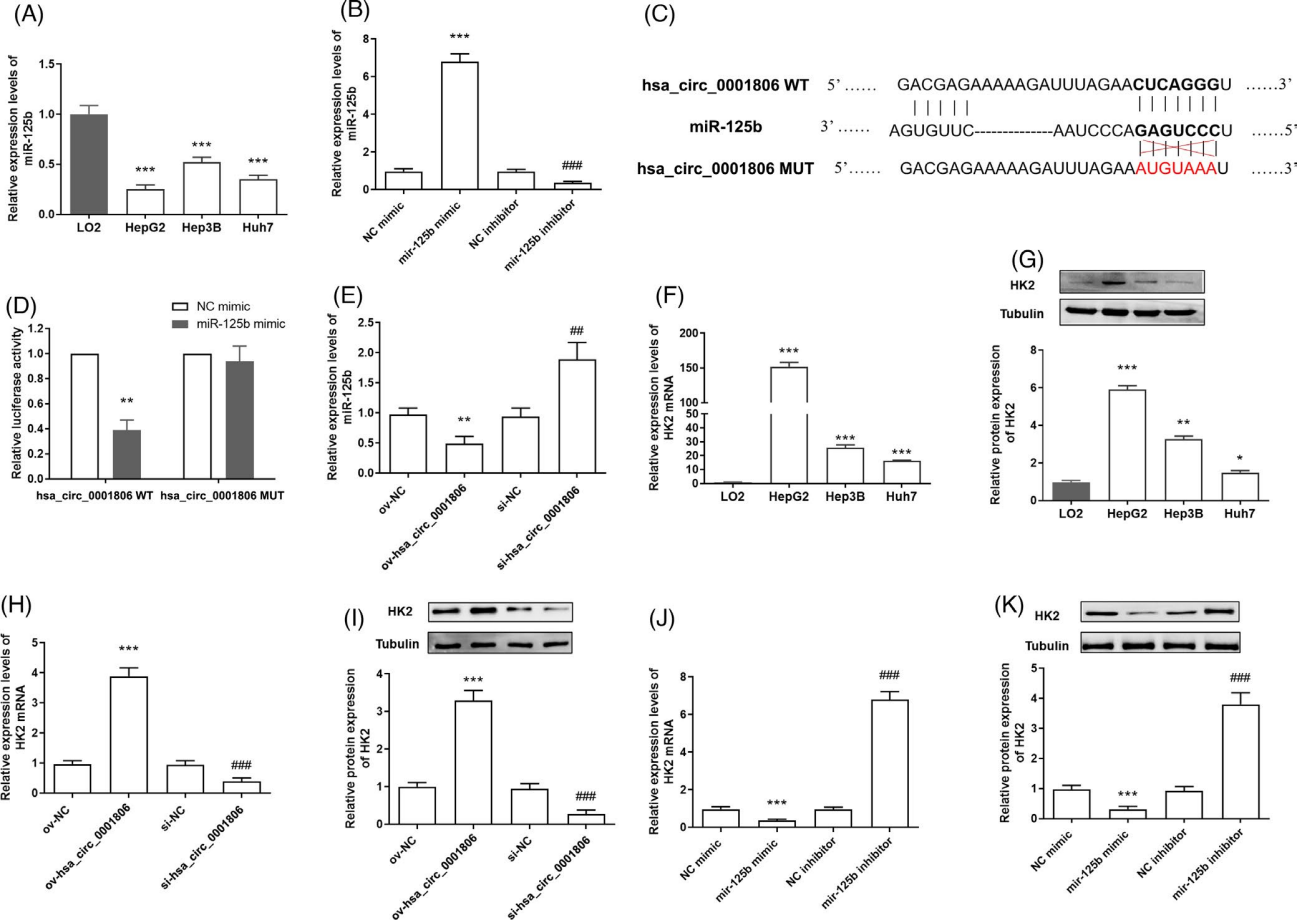
Hsa_circ_0001806 served as a sponge of miR‐125b. (A) Expression of miR‐125b in LO2, HepG2, Hep3B and Huh7 cell lines. ***p < 0.001, compared with LO2 cells. (B) Transfection efficiency of mimic and inhibitor of miR‐125b in Hep3B cells. ***p < 0.001, compared with NC mimic group; ^###^ *p* < 0.001, compared with NC inhibitor group. (C) The potential binding site of miR‐125b on hsa_circ_0001806. (D) Luciferase reporter assay for hsa_circ_0001806 WT and hsa_circ_0001806 MUT in Hep3B cells co‐transfected with miR‐125b mimic or NC mimic. **p < 0.01, compared with NC mimic‐transfected group. (E) Expression of miR‐125b in Hep3B cells after overexpression or inhibition of hsa_circ_0001806. (F and G) Relative expressions of HK2 mRNA and protein in LO2 and HCC cell lines. (H and I) Expressions of HK2 mRNA and protein in Hep3B cells after being transfected with overexpression vector or siRNA of hsa_circ_0001806. (J and K) Expressions of HK2 mRNA and protein in Hep3B cells after transfected with miR‐125b mimic or inhibitor. ***p < 0.001, compared with the ov‐NC group; ^###^ *p* < 0.001, compared with the si‐NC group

The online bioinformatics prediction tools, CircBank (http://www.circbank.cn/index.html) and BiBiServ2‐RNAhybrid (https://bibiserv.cebitec.uni‐bielefeld.de/rnahybrid), indicated that miR‐125b may serve as a target of hsa_circ_0001806. MiR‐125b was down‐regulated in HCC cells (HepG2, Hep3B and Huh7), compared with LO2 cells (Figure 3A). Mimic and inhibitor of miR‐125b were constructed and transfected into Hep3B cells to promote or suppress miR‐125b expression (Figure 3B). Furthermore, the dual‐luciferase reporter assay verified the direct binding between miR‐125b and hsa_circ_0001806 (Figure 3C,D). Meanwhile, we found that miR‐125b expression was suppressed by hsa_circ_0001806 overexpression and up‐regulated by hsa_circ_0001806 inhibition in Hep3B cells (Figure 3E). Previous studies have reported that HK2 was a key target of miR‐125b and was involved in tumour progression.^19^, ^20^ Our study verified that HK2 expression was increased in HCC cells, compared with LO2 cells (Figure 3F,G). Also, HK2 expression was promoted by hsa_circ_0001806 overexpression and the transfection of miR‐125b inhibitor and suppressed by hsa_circ_0001806 inhibition and miR‐125b overexpression in Hep3B cells (Figure 3H–K).

### 3.4 Hsa_circ_0001806 regulated the progression of HCC by regulating miR‐125b/HK2

**FIGURE 4:**
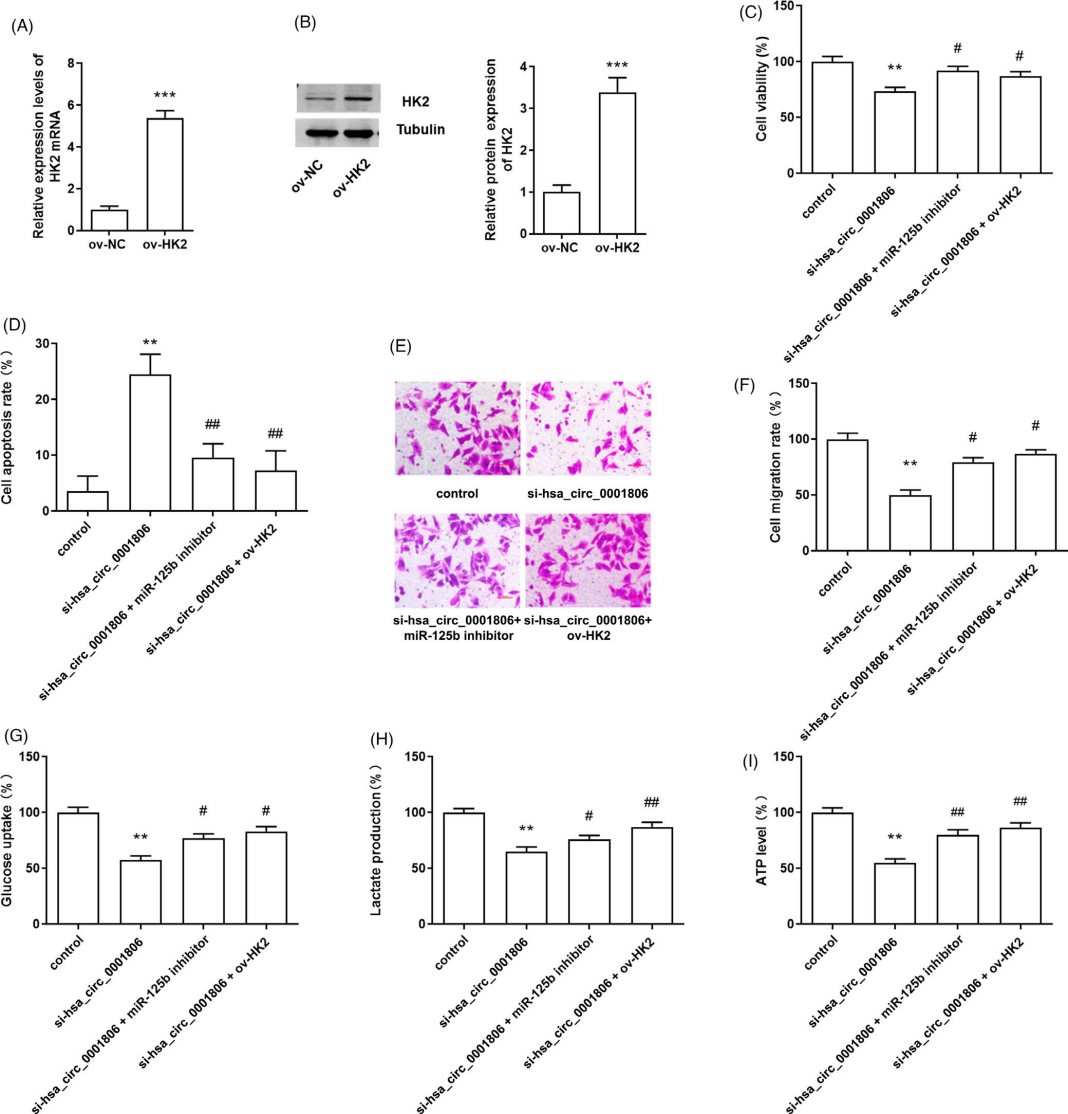
Hsa_circ_0001806 regulated the progression of HCC by mediating miR‐125b/HK2. (A and B) Transfection efficiency of the overexpression vector of HK2 in Hep3B cells. ***p < 0.001, compared with the ov‐NC transfected group. (C‐F) Cell proliferation, apoptosis and migration were measured in Hep3B cells. (G‐I) Glucose uptake, lactate and ATP production were detected in Hep3B cells. **p < 0.01, compared with the control group; ^#^ *p* < 0.05, ^##^ *p* < 0.01, compared with si‐hsa_circ_0001806 transfected group

Overexpression vector of HK2 (ov‐HK2) was constructed and transfected into Hep3B cells. The results of qRT‐PCR and Western blotting indicated that transfection of ov‐HK2 markedly up‐regulated the expressions of HK2 mRNA and protein in Hep3B cells, compared with the ov‐NC transfected group (Figure 4A,B). Furthermore, to explore the role of miR‐125b/HK2 axis in the anti‐tumour effect of hsa_circ_0001806 inhibition on HCC cells, miR‐125b inhibitor or HK2 overexpression vector was transfected into hsa_circ_0001806 silenced Hep3B cells. The results indicated that the inhibition of miR‐125b and the overexpression of HK2 both partly reversed the inhibitory effect of si‐hsa_circ_0001806 on cell viability and migration in Hep3B cells, and abated si‐hsa_circ_0001806 induced cell apoptosis (Figure 4C–F). Moreover, miR‐125b inhibition and HK2 up‐regulation increased glucose uptake, lactate production and ATP level in si‐hsa_circ_0001806‐transfected Hep3B cells (Figure 4G–I).

## 4 DISCUSSION

In this study, we found that hsa_circ_0001806 was highly expressed in HCC tissues and cells, and the increased expression of hsa_circ_0001806 was related to advanced TNM stage, lymph gland and organ metastasis, as well as poor survival, of HCC patients. This discovery was consistent with previous reports that hsa_circ_0001806 was up‐regulated in colorectal carcinoma and HCC tissues.^16^, ^18^ This suggested that hsa_circ_0001806 may serve as a molecular marker for HCC. In addition, our study indicated that the overexpression of hsa_circ_0001806 promoted cell proliferation and migration in HCC cells, while hsa_circ_0001806 inhibition suppressed cell proliferation, migration and induced cell apoptosis in HCC cells. Consistently, Cheng and colleagues discovered the elevated expression of circ_0016788 which was closely related to aggravated performance and poor outcome of HCC patients.^21^ Another study presented that circ_0004913 was dysregulated during HCC progression and the alteration of circ_0004913 could be used as an evaluation factor for HCC severity and prognosis.^22^ Liu et al. demonstrated the down‐regulation of circ_5692 in HCC tissues and cells, and circ_5692 overexpression inhibited malignance of HCC cells and the tumour growth of HCC xenograft mouse model.^23^

Accumulating evidence indicates that circRNAs functions as miRNA sponges to regulate target gene expression. In our study, we found that hsa_circ_0001806 served as a sponge of miR‐125b in HCC cells. Studies discovered that miR‐125b was down‐regulated in bladder and liver cancer and was associated with a high risk of bladder cancer, as well as poor survival rates.^19^, ^24^, ^25^ MiR‐125b overexpression suppressed cell proliferation and migration, induced cell apoptosis in bladder and liver cancer cells.^25^ It has been reported that HK2 served as a target gene of miR‐125b, which was negatively associated with miR‐125b expression in bladder cancer cells and laryngeal squamous cell carcinoma cells.^19^, ^20^ In our study, we found that hsa_circ_0001806 positively regulated HK2 expression in HCC cells. HK2 was highly expressed in several tumours, including breast cancer, ovarian cancer, gastric cancer, bladder cancer, lung cancer and hepatocellular carcinoma.^19^, ^26^, ^27^, ^28^ Also, HK2 was required for tumour initiation and maintenance in a mouse model; its inhibition suppressed the malignant behaviour of breast and lung cancer, both in vitro and in vivo.^29^ Our study identified that HK2 served as a direct target of miR‐125b in HCC cells and participated in hsa_circ_0001806‐mediated proliferation, apoptosis and migration in HCC cells.

Glycolysis provides the major energy for HCC tumour progression and is involved in cell proliferation, migration and invasion.^30^, ^31^ It is known that cancer cells exhibit aberrant metabolism and are in preference to glycolysis rather than mitochondrial oxidative phosphorylation to produce glycolytic intermediates and glucose‐dependent ATP for macromolecular biosynthesis, even under an oxygen abundant environment.^31^ HK2 is the first and initial rate‐limiting enzyme in the glycolytic pathway^27^ and catalyses glucose transforming to glucose‐6‐phosphate.^32^ The inhibition of HK2 suppressed glycolysis in HCC tumours. In addition, miR‐125b targeted to HK2 and suppressed glycolysis in laryngeal squamous cell carcinoma.^20^ The overexpression of miR‐125b promoted the dysregulation of cellular glucose metabolism by directly targeting to HK2 in human retinal pigment epithelium cells.^33^ A previous study found that astragalin suppressed HK2 through up‐regulating miR‐125b to inhibit HCC cell proliferation in vitro and in vivo.^34^ In this study, oncogenic hsa_circ_0001806 promoted glycolysis in HCC cells, while the overexpression of HK2 and inhibition of miR‐125b suppressed the inhibitory effect of hsa_circ_0001806 silencing on the glycolysis process in HCC cells.

## 5 CONCLUSION

In summary, this study investigated the role and mechanism of hsa_circ_0001806 in HCC tumours. The results verified that hsa_circ_0001806 was significantly up‐regulated in HCC tissues and cells and associated with an advanced TNM stage, lymph gland and organ metastasis and the poor prognosis of HCC patients. Moreover, hsa_circ_0001806 promoted cellular malignant behaviour and glycolysis by regulating the miR‐125b/HK2 axis, which may provide a potential therapeutic target for HCC.

## CONFLICTS OF INTEREST

The authors declare no conflicts of interest.

## Data Availability

The data used to support the findings of this study are available from the corresponding author upon request.

## References

[1] Forner A , Reig M , Bruix J . Hepatocellular carcinoma. Lancet (London, England). 2018;391(10127):1301‐1314. 10.1016/S0140-6736(18)30010-2 29307467

[2] Hartke J , Johnson M , Ghabril M . The diagnosis and treatment of hepatocellular carcinoma. Semin Diagn Pathol. 2017;34(2):153‐159. 28108047 10.1053/j.semdp.2016.12.011

[3] Sarveazad A , Agah S , Babahajian A , Amini N , Bahardoust M . Predictors of 5 year survival rate in hepatocellular carcinoma patients. J Res Med Sci. 2019;24:86. 31741658 10.4103/jrms.JRMS_1017_18 PMC6856560

[4] Reig M , Marino Z , Perello C , et al. Unexpected high rate of early tumor recurrence in patients with HCV‐related HCC undergoing interferon‐free therapy. J Hepatol. 2016;65(4):719‐726. 27084592 10.1016/j.jhep.2016.04.008

[5] Kanda T , Goto T , Hirotsu Y , Moriyama M , Omata M . Molecular mechanisms driving progression of liver cirrhosis towards hepatocellular carcinoma in chronic hepatitis B and C infections: a review. Int J Mol Sci. 2019;20(6):1358. 10.3390/ijms20061358 PMC6470669 30889843

[6] Marengo A , Rosso C , Bugianesi E . Liver cancer: connections with obesity, fatty liver, and cirrhosis. Annu Rev Med. 2016;67:103‐117. 26473416 10.1146/annurev-med-090514-013832

[7] Brunt EM , Wong VW , Nobili V , et al. Nonalcoholic fatty liver disease. Nat Rev Dis Primers. 2015;1:1‐22. 10.1038/nrdp.2015.80 27188459

[8] Cai MJ , Cui Y , Fang M , et al. Inhibition of PSMD4 blocks the tumorigenesis of hepatocellular carcinoma. Gene. 2019;702:66‐74. 30930224 10.1016/j.gene.2019.03.063

[9] Li Z , Ruan Y , Zhang H , Shen Y , Li T , Xiao B . Tumor‐suppressive circular RNAs: mechanisms underlying their suppression of tumor occurrence and use as therapeutic targets. Cancer Sci. 2019;110(12):3630‐3638. 31599076 10.1111/cas.14211 PMC6890437

[10] Sonja P , Sabine M . RNA circularization strategies in vivo and in vitro. Nucleic Acids Res. 2015;43(4):2454‐2465. 25662225 10.1093/nar/gkv045 PMC4344496

[11] Sang Y , Chen B , Song X , et al. circRNA_0025202 regulates tamoxifen sensitivity and tumor progression via regulating the miR‐182‐5p/FOXO3a axis in breast cancer. Mol Ther. 2019;27(9):1638‐1652. 31153828 10.1016/j.ymthe.2019.05.011 PMC6731174

[12] Yu J , Yang M , Zhou B , et al. CircRNA‐104718 acts as competing endogenous RNA and promotes hepatocellular carcinoma progression through microRNA‐218‐5p/TXNDC5 signaling pathway. Clini Sci. 2019;133(13):1487‐1503. 10.1042/CS20190394 31278132

[13] Wang M , Yu F , Li P . Circular RNAs: characteristics, function and clinical significance in hepatocellular carcinoma. Cancers. 2018;10(8):258. 10.3390/cancers10080258 PMC6116001 30072625

[14] Huang W , Fang K , Chen TQ , et al. circRNA circAF4 functions as an oncogene to regulate MLL‐AF4 fusion protein expression and inhibit MLL leukemia progression. J Hematol oncol. 2019;12(1):103. 31623653 10.1186/s13045-019-0800-z PMC6798510

[15] Wang Y , Li Z , Xu S , Guo J . Novel potential tumor biomarkers: circular RNAs and exosomal circular RNAs in gastrointestinal malignancies. J Clin Lab Anal. 2020;34(7):e23359. 32419229 10.1002/jcla.23359 PMC7370736

[16] Qiu L , Wang T , Ge Q , et al. Circular RNA signature in hepatocellular carcinoma. J Cancer. 2019;10(15):3361‐3372. 31293639 10.7150/jca.31243 PMC6603403

[17] Wu J , Liu S , Xiang Y , Qu X , Xie Y , Zhang X . Bioinformatic analysis of circular RNA‐Associated ceRNA network associated with hepatocellular carcinoma. Biomed Res Int. 2019;2019:8308694. 31886256 10.1155/2019/8308694 PMC6926424

[18] Sun J , Liu J , Zhu Q , Xu F , Kang L , Shi X . Hsa_circ_0001806 acts as a ceRNA to facilitate the stemness of colorectal cancer cells by increasing COL1A1. Onco Targets Ther. 2020;13:6315‐6327. 32636650 10.2147/OTT.S255485 PMC7335295

[19] Liu S , Chen Q , Wang Y . MiR‐125b‐5p suppresses the bladder cancer progression via targeting HK2 and suppressing PI3K/AKT pathway. Hum Cell. 2020;33(1):185‐194. 31605287 10.1007/s13577-019-00285-x

[20] Hui L , Zhang J , Guo X . MiR‐125b‐5p suppressed the glycolysis of laryngeal squamous cell carcinoma by down‐regulating hexokinase‐2. Biomed Pharmacother. 2018;103:1194‐1201. 29864898 10.1016/j.biopha.2018.04.098

[21] Cheng F , Wang L , Zhang J . Circular RNA 0016788 displays as a biomarker for tumor progression and poor prognosis in surgical hepatocellular carcinoma patients. J Clin Lab Anal. 2020;34(7):e23300. 32319701 10.1002/jcla.23300 PMC7370714

[22] Li X , Yang J , Yang X , Cao T . Dysregulated circ_0004913, circ_0008160, circ_0000517, and their potential as biomarkers for disease monitoring and prognosis in hepatocellular carcinoma. J Clin Lab Anal. 2021;35(6):e23785. 34018640 10.1002/jcla.23785 PMC8183933

[23] Liu Z , Yu Y , Huang Z , et al. CircRNA‐5692 inhibits the progression of hepatocellular carcinoma by sponging miR‐328‐5p to enhance DAB2IP expression. Cell Death Dis. 2019;10(12):900. 31776329 10.1038/s41419-019-2089-9 PMC6881381

[24] Inamoto T , Uehara H , Akao Y , et al. A panel of MicroRNA signature as a tool for predicting survival of patients with urothelial carcinoma of the bladder. Dis Markers. 2018;2018:5468672. 30026881 10.1155/2018/5468672 PMC6031086

[25] Hua S , Quan Y , Zhan M , Liao H , Li Y , Lu L . miR‐125b‐5p inhibits cell proliferation, migration, and invasion in hepatocellular carcinoma via targeting TXNRD1. Cancer Cell Int. 2019;19:203. 31384178 10.1186/s12935-019-0919-6 PMC6668076

[26] Li LQ , Yang Y , Chen H , Zhang L , Pan D , Xie WJ . MicroRNA‐181b inhibits glycolysis in gastric cancer cells via targeting hexokinase 2 gene. Cancer Biomark. 2016;17(1):75‐81. 27314295 10.3233/CBM-160619 PMC13020476

[27] DeWaal D , Nogueira V , Terry AR , et al. Hexokinase‐2 depletion inhibits glycolysis and induces oxidative phosphorylation in hepatocellular carcinoma and sensitizes to metformin. Nat Commun. 2018;9(1):446. 29386513 10.1038/s41467-017-02733-4 PMC5792493

[28] Suh DH , Kim MA , Kim H , et al. Association of overexpression of hexokinase II with chemoresistance in epithelial ovarian cancer. Clin Exp Med. 2014;14(3):345‐353. 23949336 10.1007/s10238-013-0250-9

[29] Patra KC , Wang Q , Bhaskar PT , et al. Hexokinase 2 is required for tumor initiation and maintenance and its systemic deletion is therapeutic in mouse models of cancer. Cancer Cell. 2013;24(2):213‐228. 23911236 10.1016/j.ccr.2013.06.014 PMC3753022

[30] Vaupel P , Schmidberger H , Mayer A . The Warburg effect: essential part of metabolic reprogramming and central contributor to cancer progression. Int J Radiat Biol. 2019;95(7):912‐919. 30822194 10.1080/09553002.2019.1589653

[31] Li Q , Pan X , Zhu D , Deng Z , Jiang R , Wang X . Circular RNA MAT2B promotes glycolysis and malignancy of hepatocellular carcinoma through the miR‐338‐3p/PKM2 axis under hypoxic stress. Hepatology. 2019;70(4):1298‐1316. 31004447 10.1002/hep.30671

[32] Lis P , Dyląg M , Niedźwiecka K , et al. The HK2 dependent "warburg effect" and mitochondrial oxidative phosphorylation in cancer: targets for effective therapy with 3‐bromopyruvate. Molecules. 2016;21(12):1730. 10.3390/molecules21121730 PMC6273842 27983708

[33] Liu G , Zhang CD , Wang J , Jia WC . Inhibition of the oxidative stress‐induced miR‐125b protects glucose metabolic disorders of human retinal pigment epithelium (RPE) cells. Cell Mol Biol (Noisy‐le‐grand). 2018;64(4):1‐5. 10.14715/cmb/2018.64.4.1 29631677

[34] Li W , Hao J , Zhang L , Cheng Z , Deng X , Shu G . Astragalin reduces hexokinase 2 through Increasing miR‐125b to Inhibit the proliferation of hepatocellular carcinoma cells in vitro and in vivo. J Agric Food Chem. 2017;65(29):5961‐5972. 28654261 10.1021/acs.jafc.7b02120

